# Nod-Like Receptors in Host Defence and Disease at the Epidermal Barrier

**DOI:** 10.3390/ijms22094677

**Published:** 2021-04-28

**Authors:** Judit Danis, Mark Mellett

**Affiliations:** 1 MTA-SZTE Dermatological Research Group, Eötvös Loránd Research Network, Korányi Fasor 6, 6720 Szeged, Hungary; 2 HCEMM-USZ Skin Research Group, University of Szeged, Korányi Fasor 6, 6720 Szeged, Hungary; 3 Department of Medical Genetics, University of Szeged, Somogyi B u 4, 6720 Szeged, Hungary; 4 Department of Dermatology, University Hospital Zürich (USZ), University of Zürich (UZH), Raemistrasse 100, 8091 Zürich, Switzerland; Mark.Mellett@usz.ch; 5 Faculty of Medicine, University of Zürich, 8091 Zürich, Switzerland

**Keywords:** NLRs, skin, keratinocyte, inflammasome, skin disease

## Abstract

The nucleotide-binding domain and leucine-rich-repeat-containing family (NLRs) (sometimes called the NOD-like receptors, though the family contains few bona fide receptors) are a superfamily of multidomain-containing proteins that detect cellular stress and microbial infection. They constitute a critical arm of the innate immune response, though their functions are not restricted to pathogen recognition and members engage in controlling inflammasome activation, antigen-presentation, transcriptional regulation, cell death and also embryogenesis. NLRs are found from basal metazoans to plants, to zebrafish, mice and humans though functions of individual members can vary from species to species. NLRs also display highly wide-ranging tissue expression. Here, we discuss the importance of NLRs to the immune response at the epidermal barrier and summarise the known role of individual family members in the pathogenesis of skin disease.

## 1. Introduction

Innate immunity relies on the recognition of evolutionarily conserved microbe-specific molecules, termed pathogen-associated molecular patterns (PAMPs). Germline encoded pattern recognition receptors (PRRs) expressed on the cell surface, endosomes or in the cytosol detect and respond to these PAMPs. Although, the domains of these PRRs are highly conserved, extensive species-specific expansions and domain shuffling result in an advantage to an organism living in pathogen-rich environments. The PRRs expressed by mammalian cells are Toll-like receptors (TLRs), the NOD-like receptors (NLRs), AIM2-like receptors (ALRs), RIG-like receptors (RLRs) and C-type lectin receptors (CLRs), with each family member recognizing specific molecular signatures [1]. Two of these families of PRRs are conserved from early invertebrates to mammals: the transmembrane TLRs and the intracellular NLRs [2,3].

Our skin acts as a sentinel organ, determining when and how to respond to a broad range of environmental insults during both homeostatic and pathologic situations. The skin forms a physical barrier through the cornified envelope of stratum corneum and via tight-junctions in lower layers, a chemical barrier by maintaining an acidic pH and antimicrobial peptide expression and finally, there is the immunologic barrier formed by keratinocytes and infiltrating immune cells of both the innate and adaptive immune systems [4]. These layers of barriers interact with each other to protect the organism from harmful stimuli. Keratinocytes are the main cell type of the epidermis and as immunocompetent cells are implicated in the protection against harmful threats, by the expression of a wide range of PRRs, including TLRs and NLRs [5,6,7]. The activation of PRRs induces keratinocytes to express antimicrobial peptides and immune mediators, which promote the recruitment of professional immune cells [4]. Murine and human TLRs in skin biology have been discussed elsewhere [5,6], here we will focus on the NLR family and discuss its role in the immune defence in the skin.

**Figure 1:**
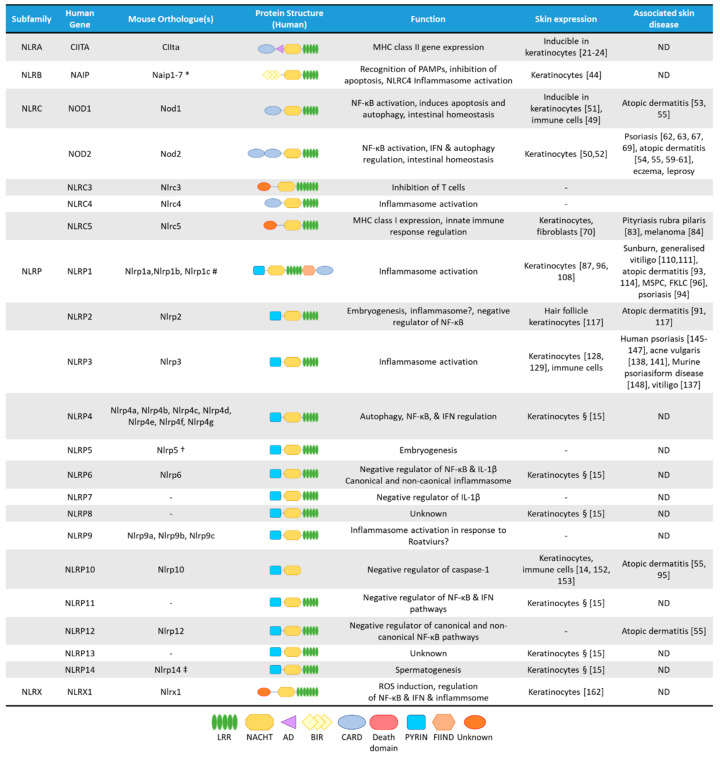
The NLR superfamily. Schematic depicting the five subfamilies of human NLRs and their mouse orthologues. Additionally, depicted are protein structural domains, the known function of the NLRs, their expression in skin cells and association with skin diseases. AD, Acidic transactivating domain, BIR, baculovirus IAP repeat, CARD, caspase-activation and recruitment domain, FIIND, Function-to-find domain, FKLC, familial keratosis lichenoides chronica, IFN, Interferon, LRR, Leucine-rich repeats, MSPC, multiple self-healing palmoplantar carcinoma, NACHT, a domain found in NAIP, CIITA, HET-E and TEP1, ND, not determined. * Murine Naip3 lacks a NACHT-LRR and contains three BIR domains, Naip4 lacks a NACHT-LRR and contains one BIR domain, Naip5 & Naip6 lack an LRR region. # Murine Nlrp1a lacks a PYRIN, Nlrp1b lacks a PYRIN and FIIND, Nlrp1c lacks PYRIN, FIIND and CARD domains. † Murine Nlrp5 lacks a PYRIN domain. ‡ Murine Nlrp14 lacks a PYRIN domain. § Based on mRNA expression assessed by Reverse Transcriptase-PCR in human primary keratinocytes [15].

NLRs are cytosolic receptors widely identified in non-vertebrates and vertebrates, and have functional analogues, the R-proteins, in plants [8]. The number of NLR genes differ from species to species: humans express 23 NLR family members, while mice have at least 34 NLR paralogues (Figure 1) [1,8]. NLRs are multi-domain containing proteins, comprising of a *C*-terminal domain with a series of leucine rich repeats (LRRs), a central nucleotide-binding NACHT domain, and an *N*-terminal effector domain [9]. The *N*-terminal domain is variable, and NLRs are divided into five subfamilies based on their distinctive *N*-terminal domain: NLRAs that have an acidic activation domain, NLRBs that possess a baculovirus inhibitor of apoptosis repeat (BIR)-like domain, NLRCs that feature a caspase activation and recruitment domain (CARD) or a Death domain (DD), and the NLRP subfamily that contain a PYRIN domain [9]. The NLRX subfamily contains one member, and its nomenclature derives from an uncharacterized *N*-terminal domain that lacks homology with other NLR effector domains.

In the cytosol, NLRs remain in an auto-inhibitory state. The LRR domains are thought to be responsible for ligand binding but this has not been experimentally shown to date for most NLRs and this dearth of evidence has led to the belief that mammalian LRRs might not have necessarily retained this function [10,11]. The LRR region also maintains the NLR in an auto-inhibitory state, as demonstrated by the crystal structure of NLRC4, where the LRR obstructs the NACHT domain [12]. The NACHT domain possesses dNTPase activity, which governs the ATP-dependent oligomerisation. Although, the NACHT domain controls oligomerisation, additionally ligand-binding can occur in this region. Upon activation, the *N*-terminal domain activates distinctive downstream signalling cascades resulting in an inflammatory response. This innate immune response also serves to influence the adaptive arm of the immune system [1]. Despite subfamilies sharing the same domain, individual members can elicit different downstream effects. For example, the NLRC family that contains a CARD domain, induces inflammasome activation, regulates nuclear factor κ-light-chain-enhancer of activated B cells (NF-κB) or type I interferon (IFN) signalling pathways or engages in transcriptional regulation [13].

**Figure 2:**
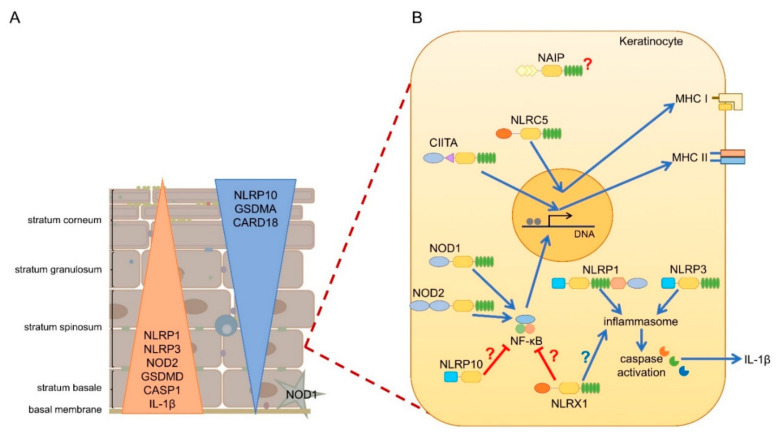
Expression and function of NLRs in human epidermis. (**A**) While inflammasome forming (NLRP1, NLRP3) and pro-inflammatory (NOD1, NOD2) NLRs are expressed in basal layers of the epidermis along with other pro-inflammatory genes, anti-inflammatory NLRs (NLRP10) and inflammasome inhibiting genes (CARD18) are rather enriched in the upper layers of the epidermis. (**B**) CIITA and NLRC5 are inducible in keratinocytes by IFN-γ and regulates MHC II and MHC I expression, respectively. NOD1 and NOD2 is activated by bacterial products, leading to NF-κB activation and inflamma Table 1. and NLRP3 are both forming inflammasomes in keratinocytes leading to IL-1β secretion NAIP, NLRP10 and NLRX1 are expressed in keratinocytes, however their functions in keratinocytes are not described so far. NLRP10 and NLRX1 were shown to inhibit NF-κB activity in professional immune cells and regulate inflammasome activation, however, whether these functions are dominant in keratinocytes is unknown.

mRNA expression of most NLRs are found in the skin, but since NLR activation is a very complex process, and some NLRs are characterized by unique cell-type specific features, without their functional evaluation in keratinocytes, their functions cannot be clearly addressed. Cornification of keratinocytes also affects the expression of NLRs and their interacting partners (Figure 2A) [14]. Here, we will summarize the current knowledge on epidermal NLR expression and functions and their potential contribution to skin disease.

## 2. The NLRA Subfamily

The NLRA subfamily comprises of a sole member, namely: the Class II Major Histocompatibility Complex Transactivator (CIITA), which contains an *N*-terminal acidic transactivation domain, but also a CARD domain, a nuclear localization signal and four LRRs [16]. CIITA has been recognized as the “master regulator” of Major Histocompatibility Complex (MHC) class II molecule (MHC-II) expression since it controls the differential expression of MHC-II genes [17]. CIITA also plays a role in human MHC class I (MHC-I) expression, a function that is not observed in mice [18]. CIITA lacks a DNA-binding domain but controls transcription by recruiting the transcription machinery, including TFIID and TFIIB [19,20]. It also induces phosphorylation of RNA polymerase I and enlists the chromatin remodelling coactivators [19,20].

CIITA is a founding member of the NLR protein family, but for a long time, it remained detached from the rest of the protein family as the only transcriptional regulator. Although, MHC-II is typically expressed by professional antigen-presenting cells, such as dendritic cells (DCs), B cells, macrophages, and thymic epithelial cells, it is long known that expression can also be induced in keratinocytes by IFN-γ [21,22,23,24]. Moreover, keratinocytes express MHC-II in a variety of skin disorders, including psoriasis, allergic contact dermatitis, and atopic dermatitis, which accompanies infiltration of activated T cells [25,26].

In mice and humans, CIITA expression is regulated by three and four distinct promoters, respectively, resulting in different isotypes [16,22]. Human keratinocytes mainly express type IV CIITA transcribed from promoter IV upon IFN-γ stimulation [22], which can be further induced by interleukin (IL)-18 [27], which subsequently leads to MHC-II expression [22]. MHC-II expressing human keratinocytes have been reported to present *Mycobacterium leprae* antigens (hsp65) and induce the proliferation of Th_1_ cells, indicating that human keratinocytes can process and present some intact protein antigens [28], however, they fail to activate naïve T-cells [29,30,31]. In contrast, mouse keratinocytes are unable to present intact proteins to specific T cells [30]. Although keratinocytes were shown to be able to express CD86 costimulatory molecules [32], generally they do not express adequate levels of CD80/CD86 [33,34,35], which may explain their inability to properly activate resting or naïve T cells. 

## 3. The NLRB Subfamily

Similarly to NLRA, the NLRB subfamily contains a single member; the neuronal apoptosis inhibitory protein (NAIP, also called NLRB1 or BIRC1) [9]. NAIP contains a BIR-like domain in its *N*-terminus [9]. There is a single human NAIP orthologue but mice contain at least 7 paralogues [36]. NAIP is an anti-apoptotic protein that acts by inhibiting caspase activity by activation of mitogen-activated protein kinase (MAPK) pathways [37,38,39]. NAIP exerts dual-functionality as it also forms an inflammasome with NLRC4 [40]. NAIP proteins directly interact with bacterial PAMPs, including flagellin. Human NAIP also binds to the T3SS needle protein of the bacterial type III secretion systems [41]. NAIPs then recruit NLRC4 as an adaptor to induce caspase-1 activation, which it does by direct CARD:CARD interactions [42,43].

According to the Human Protein Atlas, NAIP is expressed in various cell types, including keratinocytes [44]. However, compared to other NLRs the role of NAIP in the skin is poorly studied so far. NLRC4 has not been reported to be expressed in the skin, so NAIP/NLRC4 inflammasome activation may be unlikely.

## 4. The NLRC Subfamily

The NLRC subfamily is characterized by the presence of an *N*-terminal CARD-domain and is the second largest subfamily of NLRs, consisting of six members: nucleotide oligomerization domain 1 (NOD1/NLRC1), NOD2 (NLRC2), NLRC3, NLRC4 and NLRC5 [45], of which NOD1, NOD2 and NLRC5 are expressed in the skin and keratinocytes, to varying extents [46].

### 4.1. NOD1 and NOD2

NOD1 and NOD2 are prototypic PRRs and recognize intracellular bacterial peptidoglycans motifs, having one (NOD1) or two (NOD2) *N*-terminal oligomerization CARD domains [47]. Both receptors bind to the membrane of early endosomes and oligomerise upon ligand binding, leading to the activation of downstream signalling of NF-κB and MAPK pathways [45], or the activation of autophagy independently of NF-κB [48].

Both NOD1 and NOD2 are expressed in the skin, although NOD1 is mainly expressed by skin-homing immune cells [49], while NOD2 expression is comparable in both immune cells and basal keratinocytes (Figure 2A) [50]. In human keratinocytes, NOD1 expression is induced by IFN-γ and was shown to activate IL-8 expression in response to *Pseudomonas aeruginosa* [51]. NOD2 mediates beta-defensin-2 expression in keratinocytes subsequent to muramyl dipeptide exposure [52].

#### Skin Diseases Associated with NOD1 and NOD2 Functions

Genetic variants for both *NOD1* and *NOD2* (and also *NLRP12*) are linked to atopic dermatitis [53,54,55]. Atopic dermatitis is the most common inflammatory skin disease [56] and is characterized by impaired skin barrier function, reduced expression of antimicrobial peptides and Th_2_-driven inflammation. Atopic dermatitis is also accompanied by a defect in innate immune receptor functions and colonization by *Staphylococcus aureus (S. aureus)* [4,57]. It is thought, that the “leaky” epithelial barrier promotes allergen sensitization and susceptibility to microbial colonization [58]. In atopic dermatitis cohorts *NOD1* SNPs were associated with increased IgE levels, and more weakly with atopic dermatitis [53], while a polymorphic *NOD2* allele was associated with an almost 2-fold risk of atopic dermatitis [54]. Furthermore, a missense variant of NOD2 and a rare NOD1 haplotype were observed more frequently in patients with atopic dermatitis than in control subjects [55]. Functional deficiencies in NOD2 might result in a higher risk of *S. aureus* colonization, often observed in atopic dermatitis. *Nod2*-deficient mice display impaired clearance of *S. aureus* after subcutaneous or intraperitoneal infection [59,60]. Human data show *S. aureus*-induced activation of NOD2 in keratinocytes results in increased expression of IL-17C, a pathway that might be dysregulated in atopic dermatitis [61].

Although, two missense mutations of NOD2 (R702W, G908R) and one frame-shift mutation Leu1007fsinsC (3020insC) were suggested to be genetic risk factors for psoriasis [62], there is no clear association of *NOD2* genetic variants to the disease [63]. Psoriasis pathogenesis arises from a complex interplay of the innate immune response in keratinocytes, skin-resident immune cells and infiltrating leukocytes [64]. Though, neutrophils and myeloid cells play an important role, immunopathogenesis is also driven by Th_1_ and Th_17_ cells. The development of psoriatic plaques is caused by the interaction of keratinocytes with these infiltrating immune cells, leading to uncontrolled keratinocyte proliferation and dysfunctional differentiation. However, keratinocytes can have an initiating role in disease development [65,66]. NOD2 is also highly expressed in psoriatic skin [67] and *Nod2*^–/–^ mice are more susceptible to imiquimod-induced psoriasiform disease, suggesting an inflammation-limiting role of Nod2 in murine disease. Moreover, chronic activation of NOD2 by muramyl dipeptide induces tolerance to bacterial products [68], and topical treatment with muramyl dipeptide is an effective therapy for psoriasis treatment [69]. 

### 4.2. NLRC5

NLRC5 is abundant in human skin with similar expression levels in keratinocytes to other cell types [70]. NLRC5 has the highest homology to CIITA and also contains a nuclear localization sequence. The inclusion of NLRC5 in the NLRC family is due to the presence of a CARD-like domain [71]. NLRC5 shuttles between the cytoplasm and nucleus and similar to CIITA induces the transcription of MHC class I genes in mouse and human cells [72,73,74]. Like CIITA, NLRC5 also forms an enhanceosome and domain swapping experiments showed that the DD domain of both act as transcriptional activation domains [75].

Besides its role in regulating antigen presentation, NLRC5 also plays a role in several cellular inflammatory reactions [76]. In certain cell types, NLRC5 serves as a negative regulator of NF-κB activity by blocking the phosphorylation of IKKα and IKKβ [77,78]. It was also reported to both promote and limit the antiviral type I interferon responses [77,79,80,81]. NLRC5 was also demonstrated to cooperate with the NLRP3 inflammasome in response to bacterial infection [82]. However, whether NLRC5 exerts any of these functions in the epidermis or in keratinocytes has not been studied so far.

#### Skin Diseases Associated with NLRC5 Functions

Although NLRC5 functions have not been deeply studied in keratinocytes, its role in skin immune responses is supported by a genetic study. In a family, harbouring a pityriasis rubra pilaris (PRP)-causing gain-of-function *CARD14* mutation, the clinical manifestation was milder in a family member, who also carried an additional mutation of *NLRC5*. This suggests a potential interplay of the two molecules in mediating the inflammatory response in keratinocytes [83]. Interestingly, the epigenetic modifier Protein arginine methyltransferase 5 (PRMT5) downregulates NLRC5 expression in melanoma cells, leading to a decrease of MHC-I-mediated antigen presentation. Knockdown of PRMT5 promoted MHC-I accumulation at the cell surface of melanoma cells [84]. NLRC5 is also highly expressed in keloids, fibrotic tumours in the skin that arise due to fibroblast hyperproliferation and increased expression of the extracellular matrix. Knockdown of *NLRC5* inhibits production of extracellular matrix components in keloid fibroblasts [85].

## 5. The NLRP Subfamily

The NLRP subfamily has 14 members in humans, of which NLRP7, NLRP8, NLRP11 and NLRP13 have no orthologues in mice, while other members of the family have three (NLRP1, NLRP9), or seven (NLRP4) murine orthologues (Figure 1) [86]. NLRP proteins are characterized by the presence of an *N*-terminal PYRIN domain, allowing the recruitment of the inflammasome-activating scaffold protein Apoptosis-associated speck-like protein containing a CARD (ASC) [1]. Gene-expression of most NLRPs can be detected in immune cells and in keratinocytes [15]. Besides protein expression in immune cells, NLRP1 [87], NLRP3 [88] and NLRP10 [14] are found in human skin samples, with other members being more predominantly expressed in other tissues. 

Some members of the NLRP family (NLRP1, NLRP3, NLRP6, NLRP7 and NLRP12) form inflammasomes, leading to the activation of inflammatory caspases with subsequent IL-1β processing and release via inflammatory cell death, termed pyroptosis [89]. Unlike professional immune cells, human keratinocytes do not need a priming signal to express inflammasome components, such as NLRP1, NLRP3, pro-IL-1β, ASC or pro-caspase-1 [15,87,90]. Moreover, the involvement of the NLRP subfamily to various skin diseases has been shown by association of genetic variants to inflammatory skin lesions, such as psoriasis, atopic dermatitis, and vitiligo as well as skin cancers [91,92,93,94,95,96]. 

### 5.1. NLRP1

NLRP1 was the first described member of the NLRP subfamily to form inflammasome [97]. There are three murine NLRP1 homologues to the gene: *Nlrp1a*, *Nlrp1b*, *Nlrp1c* [1,98]. NLRP1 contains an *N*-terminal PYRIN domain, a NACHT domain, LRRs, but also harbours a *C*-terminal function-to-find domain (FIIND) and a CARD domain, through which it can directly activate caspase-1, albeit association with ASC enhances this activation [99]. Interestingly, murine NLRP1 orthologues, Nlrp1a, -b and -c lack PYRIN domains [100]. Activation of NLRP1 is unique among the NLRP family, as it undergoes auto-proteolysis within the FIIND and the resulting *N*- and *C*-terminal fragments remain non-covalently associated and auto-inhibited [101]. Murine Nlrp1b was shown to be subsequently subjected to “functional degradation”, where the inhibitory *N*-terminal domain is targeted for *N*-end rule ubiquitination and proteasomal degradation, thus liberating and activating the *C*-terminal fragment, which can form an inflammasome with capase-1 [102]. Human NLRP1 was initially described to be activated by muramyl dipeptide, while the murine protein is activated by *Bacillus anthracis* lethal toxin [103,104,105] and *Toxoplasma gondii* [106]. More recently, NLRP1 was found to be activated in response to viral agonists, including dsRNA in keratinocytes, which binds directly to the NACHT-LRR region and induces ATP hydrolysis by the NACHT domain [107]. Ultraviolet B irradiation (UVB) also induces activation of the NLRP1 inflammasome in human skin and keratinocytes, while murine keratinocytes fail to activate inflammasome upon UVB exposure [87,90]. However, UVB irradiation does induce IL-1β release in murine skin, which is mediated by infiltrating professional immune cells (mainly dendritic cells) and not keratinocytes [87]. These result show the poor conservation of the NLRP1 pathway between human and mouse skin [108]. Interestingly, 3C proteases and dsRNA only activate human NLRP1, whereas Anthrax Lethal Factor metalloprotease induces cleavage of murine Nlrp1b but not the human form [109]. *T. gondii* infection and Dpp8/9 inhibition with Talabostat (Val-boroPro) commonly activated both murine and human isoforms. Interestingly, Talabostat activates CARD8 in THP-1 cell-lines but triggers NLRP1 activation in keratinocytes, despite both cell types expressing both sensors [108].

#### Skin Diseases Associated with Genetic Variants in NLRP1

The human *NLRP1* gene is highly polymorphic and GWAS studies have linked *NLRP1* SNPs to congenital toxoplasmosis, Addison’s disease (hypocortisolism and adrenal insufficiency) but also to generalized vitiligo [110,111]. Vitiligo is an autoimmune depigmenting disorder where infiltrating and skin-resident CD8+ cytotoxic T cells induce the loss of melanocytes [112].

Additionally, germline mutations that disrupt the PYRIN and LRR domains are reported to cause two skin disorders: multiple self-healing palmoplantar carcinoma (MSPC) and familial keratosis lichenoides chronica (FKLC) [96]. Interestingly, these diseases are not associated with fever, which typically accompanies inflammasome-related syndromes. PYRIN and LRR domains promote auto-inhibition of NLRP1 and mutations disturb this regulatory mechanism, lowering the threshold of NLRP1 activation. This leads to skin hyperplasia and formation of keratoacanthoma, the continuous cycles of immune clearance and inflammasome activation may promote the acquisition of oncogenic mutations that facilitate the development of squamous cell carcinoma. This is an interesting association of NLRP1 and skin cancer, reinforced by the fact that the NLRP1 agonist, UVB irradiation, is a primary risk factor for keratinocyte carcinomas [96,108,113].

Polymorphisms in *NLRP1* that could alter protein expression, can lead to a dysregulation in pathogen recognition and response in atopic dermatitis patients. In severe cases NLRP1 expression shows an inverse correlation with symptoms. The impaired wound healing and defense responses in atopic dermatitis might be caused by the downregulation of NLRP1 expression [114]. The missense variants, potentially affecting NLRP1 functions were predicted to be functionally significant in the susceptibility of atopic dermatitis [93]. 

### 5.2. NLRP2

NLRP2 has a role in embryo development but its role in inflammasome activation is less clear. NLRP2 inhibits NF-κB activation but activates caspase-1 transcription [115]. NLRP2 was reported to form inflammasomes in gingival epithelial cells but whether it is expressed or functional in epithelial skin is not elucidated [116]. 

#### Skin Diseases Associated with NLRP2 Functions

Although, NLRP2 is not highly expressed in human or mouse skin, association of decreased NLRP2 expression with early onset atopic dermatitis was described due to promoter hypermethylation in immune cells of the patients [91]. Additionally, NLRP2 and IL-1β expression was more upregulated in human hair follicle-derived keratinocytes from atopic dermatitis patients than controls [117] and it would be of interest to further explore whether the NLRP2 inflammasome plays a role in atopic dermatitis-associated inflammation.

### 5.3. NLRP3

NLRP3 is the mostly extensively studied member of the NLRP subfamily and is predominantly expressed in immune cells. NLRP3 activation in immune cells requires a two-step process with a priming signal for transcriptional induction of signalling molecules, including NLRP3 itself, followed by a second, inflammasome activating signal. Both human and murine NLRP3 inflammasome activation can be initiated by numerous signals derived from cellular damage, such as ATP release [118], potassium efflux [119], reactive oxygen species, cathepsins and microcrystals [120], but activation by microbial products has also been described [121,122]. However, due to the diversity of these agonists, it is likely that NLRP3 does not bind directly to microbial PAMPs but instead detects cellular perturbation as part of a “Guard model”, similar to the detection of Rho GTPases by Pyrin after bacterial infection [13].

Upon activation, NLRP3 undergoes a conformational change permitting NACHT domain-mediated oligomerisation. This aids recruitment of ASC to the PYRIN domain of NLRP3, and ASC further forms polymers that engages pro-caspase-1. Caspase-1 undergoes auto-cleavage and cleaves pro-IL-1β to its mature form [123]. In addition, to this “canonical” inflammasome, NLRP3 can also engage in a second “non-canonical” inflammasome. In response to intracellular lipopolysaccharide, caspase-11 (in mouse, caspase-4 and -5 in humans) undergoes oligomerization and auto-activation. Caspase-11 cleaves Gasdermin D (GSDMD), which forms pores permitting the escape of mature IL-1β and IL-18 [124]. However, these pores upset the osmotic balance between the intra- and extracellular environments triggering pyroptosis. K^+^ efflux as a result of these pores activates NLRP3 downstream of caspase-11 [125]. Caspase-1 also cleaves GSDMD, an event essential in pyroptosis, downstream of the canonical inflammasome [126]. In macrophages, GSDMD cleavage by caspase-1 results in pyroptosis, but in human primary keratinocytes GSDMD is a poor substrate of caspase-1 and rather supports secretion of IL-1β, without pyroptosis [127].

Although, NLRP1 is regarded as the principal inflammasome sensor in human keratinocytes [87,96,108] expression of NLRP3 can also be detected in basal keratinocytes (Figure 2A) [128,129]. NLRP3 activation in human keratinocytes can be initiated by various signals [128,129] but without a need for a priming signal. Human keratinocytes respond to viral RNA with caspase-1 activation and subsequent IL-1β and IL-18 release, which is dependent on NLRP3 [129]. Zhang and colleagues reported that soluble CD100 binds to PlxnB2 and activates NLRP3 in keratinocytes, leading to IL-1β and IL-18 release [130]. UVB irradiation also indirectly causes NLRP3 inflammasome activation in keratinocytes by inducing cyclobutane pyrimidine dimer formation in the DNA, and the damaged DNA can induce NLRP3 inflammasome activation, leading to IL-1β release [131]. Inflammasome activation is also indispensable for the normal wound healing processes, as demonstrated in mouse models, mainly due to macrophages and fibroblasts, not due to keratinocytes [132,133]. However, overactivation of inflammasomes has an opposing effect, and inhibits normal wound healing [134,135].

#### 5.3.1. The Role of NLRP3 in Skin Diseases

NLRP3 has been described to have indispensable role in the pathogenesis of numerous skin diseases, including acne, atopic dermatitis, urticaria (or hives), bullous pemphigoid, vitiligo and psoriasis [136,137].

NLRP3 is activated in sebocytes by *Cutibacterium acnes* (previously referred to as *Propinibacterium acnes*) [138], the prominent member of microbiota, which is thought to be responsible for acne vulgaris formation [139,140]. Moreover, genetic variants of *NLRP3* could also be linked to acne vulgaris in a Han Chinese population [141].

Although genetic predispositions of *NLRP3* have not been discovered for atopic dermatitis [57], experimental data suggests that activation of the NLRP3 inflammasome might play a role in the disease. Expression of NLRP3 and caspase-1 was significantly impaired in lesional skin of atopic dermatitis patients compared to healthy controls [88]. Allergens and atopic dermatitis triggers, such as *S. aureus*-derived compounds can also activate NLRP3 in monocytic cells [142,143], in human keratinocytes [144], and in mouse keratinocytes [15].

In human and murine psoriatic skin NLRP3 expression is upregulated [145,146]. Two *NLRP3* SNPs (rs3806265 and rs10754557) were found to be significantly related to psoriasis in a Han Chinese population [147]. In this same study, *NLRP1* SNPs were found to have no significant relation to psoriasis, though in another study in a Swedish cohort, *NLRP1* SNPs were linked to psoriasis susceptibility [94]. Interestingly, ASC shows elevated expression in psoriatic epidermis and also displays more nuclear expression compared to control skin [67]. NLRP3 inflammasome inhibitor, CP-456,773 (now renamed MCC950) significantly alleviated murine imiquimod-induced psoriasiform disease [148]. Similarly, cycloastragenol that also inhibits NLRP3-mediated pyroptosis can also decrease symptoms in imiquimod-induced skin inflammation in mice [149]. These findings suggest that targeting NLRP3 could be avenue to explore for psoriasis treatment.

#### 5.3.2. Inflammasome Activation and Epidermal Differentiation Are Interconnected

Proteins of the gasdermin (GSDM) family are not only involved in inflammasome activation and pyroptosis, but are also indispensable for normal terminal differentiation and cornification processes in the skin [14,150,151]. The most prominent member of the family is GSDMD. All GSDMs are activated by proteolytic cleavage by caspases between the *N*-terminal pore-forming and a *C*-terminal repressor domain, allowing for pore formation. GSDMs involved in inflammatory processes (i.e., GSDMD and GSDME) are expressed in basal layers of skin. However, during terminal differentiation their expression decreases in human keratinocytes along with the inflammasome components and substrate IL-1β (Figure 2A) [14]. These results suggest that human epidermal cornification is accompanied by a tight control of inflammasome activation and it is of interest to further tease out the role of this protein family in these divergent types of cell death in the skin.

### 5.4. NLRP10

Both human and murine NLRP10 are highly expressed in the epidermis and contribute to cell-autonomous responses against invasive bacteria [152]. Compared to other organs, murine epidermis was found to show the highest expression of Nlrp10 mRNA [153]. Since NLRP10 lacks the prototypical *C*-terminal leucine-rich repeats, it is thought to function as a signalling modifier. Indeed, NLRP10 positively regulates innate immune responses mediated by NOD1 upon *Shigella flexneri* infection in both epithelial cells and dermal fibroblasts by modulating p38 MAPK and NF-κB signalling [152]. Human NLRP10 inhibits ASC-mediated NF-κB activation and caspase-1 maturation of IL-1β [154]. However, immune cells from *Nlrp10* knock-out mice respond normally to inflammasome activation [155,156], suggesting a difference in function between the human and mouse proteins.

In mice, Nlrp10 has a bridging function between the innate and adaptive immune responses through DC activity. Against T-cell dependent antigens *Nlrp10*-deficient mice show no efficient antigen specific immune responses due to impaired DC responses [156], which also impairs the response to infection of the fungal pathogen *Candida albicans* [155]. The lack of connection between the adaptive and innate immune system in the absence of Nlrp10 was further shown in other models. *Nlrp10* knock-out mice had significantly decreased inflammation in induced-contact hypersensitivity models and this was accompanied by a decreased infiltration of T cells. Mice with epidermal-specific knockout of Nlrp10 expression also displayed less inflammation but no loss of infiltrating T cell numbers [157]. Interestingly, NLRP10 expression among other inflammasome regulators was strongly induced in differentiated human keratinocytes (Figure 2A) [14]. Moreover, GWAS studies also linked *NLRP10* genetic variants to atopic dermatitis [55,95], a skin disease accompanied by abnormal differentiation and decreased barrier functions [158]. These data strongly suggest a physiological role of NLRP10 in the skin, in addition to immune cells.

## 6. The NLRX Subfamily

The sole member of the NLRX family, NLRX1 contains a dissimilar and uncharacterised *N*-terminal effector domain compared to other NLRs. It also has an unusual *C*-terminus, which contains seven LRRs and a three-helix bundle [159]. Within the *N*-terminus NLRX1 contains a mitochondria-targeting sequence [160,161,162] and is involved in mitochondrial reactive oxygen species (ROS) formation [162]. Additionally, NLRX1 attenuates NF-κB and inflammasome signalling [162,163]. The regulatory effects of NLRX1 are highly cell type specific, which might be determined by the unique functional activity or metabolic profile of the given cell type [164]. NLRX1 is ubiquiteously expressed, including in keratinocytes, but its function in the skin is currently unknown.

## 7. NLRs in Human and Mouse Skin

Animal models are indispensable to study the mechanism of human diseases. The mouse represents one of our most reliable animal models, supported by results of human and murine genome sequencing, which reveal that only a couple hundreds of genes appear to be unique to one species or the other [165]. However, in the study of immune-derived skin diseases, it is challenging to translate results derived from mouse to human. There are several structural and functional differences between mouse and human skin. Human skin is thicker, with 5 to 10 cell layers in the epidermis, adhering tightly to underlying tissues, while murine skin is thinner and loose, containing only 2 to 3 cell layers in the epidermis, associated with decreased barrier function and increased absorption of murine skin [166].

In general, it is believed that murine and human keratinocytes share many common characteristics. Both mouse and human epidermis are implicated in the protection against endogenous and exogenous harmful stimuli, however, their immunological mechanics differ. While human keratinocytes express a wide range of PRRs, actively participating in immune defence against skin-invading pathogens, in murine skin a higher number of skin-resident professional immune cells perform these defence functions [87].

While there are numerous comparative analyses on TLR expression and functions in skin cells of various species, including human and murine keratinocytes [3,5,165,167,168,169,170], there is limited information on NLR function in these cell types. Both human and mouse keratinocytes were shown to express NLR proteins (Figure 1) however their activators are highly cell-type specific, thus their functions might differ from professional immune cells. The few studies comparing NLR expression and function in human and mouse keratinocytes or skin have shown differences in their functions with significantly lower expression in murine keratinocytes than in human cells (Table 1). 

CIITA is expressed in both human and mouse keratinocytes regulating MHC-II expression. However, while human keratinocytes are able to use the expressed MHC molecules for antigen presentation to T-cells, murine keratinocyte are unable to do so [29,30,31]. NLRP1 is expressed and functional in human keratinocytes, its mouse orthologues are not expressed in keratinocytes rather in skin-homing immune cells [87]. Interestingly, intact skin of both human and mouse produce the same mediators upon the NLRP1 activating UVB exposure, but while in humans the process is mediated by NLRP1 in keratinocytes, in mouse skin the immune cells are responsible for this phenomenon [87]. Similarly, NLRP3 is also expressed and functional in human keratinocytes [128,129], but not in murine epidermis [171]. In human cells, NLRP10 regulates inflammasome activation [152,154], while *Nlrp10* knock-out mice respond normally to inflammasome activation [155,156], suggesting a difference in function between the human and mouse proteins. 

Not only are NLRs differentially expressed in skin samples, but other components of inflammasome activation also show differences between species. Especially during terminal differentiation expression of inflammasome inhibitors, including CARD18, increases [14,172,173], while expression of inflammasome members, pore-forming GSDMs and inflammasome substrate IL-1β decreases (Figure 2A) [14]. 

Expression of GSDMA also increases in differentiated keratinocytes, suggesting a function for GSDMA during cornification (Figure 2A) [14,150]. While humans have one *GSDMA* gene, mice have three orthologues, *Gsdma*, *Gsdma2*, *Gsdma3*. Knock-out mouse models showed the involvement of Gsdma3 and Gsdma in terminal differentiation of keratinocytes and hair follicle formation, respectively. Mutations in *Gsdma3* were also linked to alopecia in mice, however these results have so far not been corroborated in human studies [151].

This would indicate that results obtained in mouse models require careful interpretation, and comparison with human data, to draw precise conclusions on NLR functions. Thus far, most of our knowledge on the functions NLRs in skin biology have come from mouse models. However, mice and humans have striking differences in their skin structure and NLR expression and functions between human and murine keratinocytes.

**Table 1:** Comparison of human and murine NLR functions.

Human Gene	Murine Gene	Function in Human Keratincoytes/Skin	Function in Murine Keratinocytes/Skin
CIITA	CIIta	Inducible in keratinocytes [21,22,24] Regulates MHC-II expression and presentation of intact proteins to T-cells [22,27,28],	Inducible in keratinocytes [23,30]
NOD2	Nod2	Gene variants associated with atopic dermatitis [53,54,55] Deficiency promotes *Staphylococcus aureus* colonisation [61]. Upregulated in psoriasis [67]	Nod2^–/–^ are unable to clear *Staphylococcus aureus* infection [59,60] Nod2^–/–^ mice are susceptible to imiquimod-induced psoriasiform disease [68]
NLRP1	Nlrp1a, Nlrp1b, Nlrp1c #	Expressed in keratinocytes [87] dsRNA and UVB induced activation in keratinocytes [87,90,107]	Not expressed in keratinocytes [87] UVB induced activation in skin-homing immune cells [87]
NLRP3	Nlrp3	Expressed and activated in keratinocytes [128,129] Activated in sebocytes by *Cutibacterium acnes* [138] Activated in keratinocytes by *Staphylococcus aureus* [144] Upregulated in psoriasis [145]	Expressed mainly in skin-homing immune cells [132,133] Role in wound healing [132,133] Activated in keratinocytes by *Staphylococcus aureus* [15] Upregulated in psoriasiform disease [146,148,149]
NLRP10	Nlrp10	Expressed in differentiated keratinocytes [14,152] Gene variants associated with atopic dermatitis [55,95] Regulates inflammasome activation [152,154]	Expressed in differentiated keratinocytes [14,153] Plays a role in contact hypersensitivity [157] Nlrp10^–/–^ mice exhibit normal inflammasome activation [155,156]

## 8. Conclusions

Since the discovery of NLRs, our knowledge has rapidly increased on their functions and regulation, especially in professional immune cells. Several NLRs are expressed in skin, while evidence is lacking for the expression of others. Skin is the first line of defence against invading pathogens. Keratinocytes are the main cell type of the epidermis and as immunocompetent cells are implicated in the protection against harmful stimuli, partially due through NLR activation. Moreover, terminal differentiation of keratinocytes also affects the expression of NLRs and their interacting partners. This dichotomy of expression of individual NLRs in proliferating and differentiating keratinocytes is intriguing and warrants further investigation. It should be noted that mRNA levels of most NLRs are found in skin and since some inflammasome components require a “priming” step, it cannot be ruled out that in particular inflammatory contexts expression of certain NLRs are not enhanced. NLR activation is a very complex process, moreover, some NLRs are characterized by unique features which can be also dependent on the cell-type. Therefore, without functional evaluation of NLRs in keratinocytes, their functions cannot be clearly addressed. However, as the field grows, a better understanding on specific NLR functions in the skin and an appreciation of their contribution to skin disease can be expected in the near future.

## Data Availability

Not applicable.
